# Direct Oral Anticoagulants for Pulmonary Embolism: Importance of Anatomical Extent

**DOI:** 10.1055/s-0037-1615251

**Published:** 2018-01-08

**Authors:** Marjolein P. A. Brekelmans, Harry R. Büller, Michele F. Mercuri, Walter Ageno, Cathy Z. Chen, Alexander T. Cohen, Nick van Es, Michael A. Grosso, Andria P. Medina, Gary Raskob, Annelise Segers, Thomas Vanassche, Peter Verhamme, Philip S. Wells, George Zhang, Jeffrey I. Weitz

**Affiliations:** 1 Department of Vascular Medicine, Academic Medical Center, Amsterdam, The Netherlands; 2 Clinical Development, Daiichi Sankyo Pharma Development, Edison, New Jersey, United States; 3 Department of Clinical and Experimental Medicine, University of Insubria, Varese, Italy; 4 Global Medical Affairs, Daiichi Sankyo Inc., Parsippany, New Jersey, United States; 5 Department of Haematological Medicine, Guy's and St Thomas' Hospitals, King's College London, London, United Kingdom; 6 Department of Medicine, University of Oklahoma Health Sciences Center, Oklahoma City, Oklahoma, United States; 7 College of Public Health, University of Oklahoma Health Sciences Center, Oklahoma City, Oklahoma, United States; 8 Itreas BV, Amsterdam, The Netherlands; 9 Vascular Medicine and Haemostasis, University of Leuven, Leuven, Belgium; 10 Department of Medicine, University of Ottawa, Ottawa Hospital Research Institute, Ottawa, Canada; 11 Thrombosis and Atherosclerosis Research Institute and McMaster University, Hamilton, Ontario, Canada

**Keywords:** pulmonary embolism, venous thromboembolism, right ventricular dysfunction, oral anticoagulants, anatomical extent

## Abstract

Pulmonary embolism (PE) studies used direct oral anticoagulants (DOACs) with or without initial heparin. We aimed to (1) evaluate if PE patients benefit from initial heparin; (2) describe patient characteristics in the DOAC studies; and (3) investigate whether the anatomical extent of PE correlates with N-terminal pro-brain natriuretic peptide (NT-proBNP) levels, cause of PE, and recurrence rate. Our methods were (1) an indirect meta-analysis comparing the recurrence risk in DOAC-treated patients with or without initial heparin to those patients given heparin/vitamin K antagonist (VKA). (2) To compare the PE studies, information was extracted on baseline characteristics including anatomical extent. (3) The Hokusai-VTE study was used to correlate anatomical extent of PE with NT-proBNP levels, causes of PE, and recurrent venous thromboembolism (VTE). The meta-analysis included 11,539 PE patients. The relative risk of recurrent VTE with DOACs versus heparin/VKAs was 0.8 (95% confidence interval [CI]: 0.6–1.1) with heparin lead-in and 1.1 (95% CI: 0.8–1.5) without heparin. In the DOAC studies, the proportion of patients with extensive PE varied from 24 to 47%. In Hokusai-VTE, NT-proBNP was elevated in 4% of patients with limited and in over 60% of patients with extensive disease. Cause of PE and anatomical extent were not related. Recurrence rates increased from 1.6% with limited to 3.2% with extensive disease in heparin/edoxaban-treated patients, and from 2.4 to 3.9% in heparin/warfarin recipients. In conclusion, indirect evidence suggests a heparin lead-in before DOACs may be advantageous in PE. Anatomical extent was related to elevated NT-proBNP and outcome, but not to PE cause.

## Introduction

Direct oral anticoagulants (DOACs) are at least as effective as vitamin K antagonists (VKAs) for treatment of venous thromboembolism (VTE), but are more convenient to administer and are associated with less bleeding. 1 Because of these attributes, current guidelines give preference to the DOACs over heparin/VKAs for VTE treatment in patients without active cancer. 2

Studies comparing DOACs with heparin/VKAs have either started the DOAC after a mandatory heparin lead-in of at least 5 days, or have adopted an all-oral DOAC regimen. 3 4 5 6 7 8 These approaches have not been directly compared. In all studies, the comparator was standard of care at that time, consisting of a minimum 5-day course of heparin overlapping with a VKA targeted at an international normalized ratio (INR) of 2.0 to 3.0.

Although all-oral regimens of DOACs are available, clinicians are often reluctant to forgo heparin treatment in patients with pulmonary embolism (PE), particularly in those with computed tomographic (CT) evidence of extensive disease and/or right ventricular enlargement. 9 10 11

The first objective of the current analysis was to evaluate whether PE patients may benefit from initial heparin therapy by indirectly comparing the rate of recurrent VTE in studies with and without a mandatory heparin lead-in. The second objective was to describe the characteristics of PE patients across the studies to assess whether relevant differences exist. The third objective was to better understand whether the anatomical extent of PE correlates with the presence of right ventricular dysfunction (RVD), as evidenced by an elevated serum N-terminal pro-brain natriuretic peptide (NT-proBNP) level, the cause of PE, and the rate of recurrence. For the third purpose, we used data from Hokusai-VTE, because this was the only study that prospectively assessed all these variables. 8 12

## Methods

### Objective 1: Recurrent VTE with or without Mandatory Heparin Lead-in

A meta-analysis of phase 3 randomized controlled trials comparing DOACs with heparin/VKAs for treatment of acute symptomatic VTE was performed. The selection and identification of these studies were part of a previously published study. 13 We extracted data on the PE patients from RE-COVER, RE-COVER II, EINSTEIN-PE, AMPLIFY, and Hokusai-VTE. In this analysis, we focused on recurrent VTE, which was defined in a similar fashion in these studies, and compared the rates of recurrence in patients treated with heparin overlapping with a VKA and DOACs with or without a mandatory heparin lead-in.

We calculated relative risks (RR) and corresponding 95% confidence intervals (CI) for recurrent VTE; results were combined using the Mantel-Haenszel method and compared using the DerSimonian and Laird random-effects model. We assessed and quantified statistical heterogeneity across the studies using the Cochran's Q and I ^2^ statistics. The analysis was performed using Review Manager, version 5.2 (Copenhagen, The Nordic Cochrane Centre, The Cochrane Collaboration, 2012).

### Objective 2: Patient Characteristics in the DOAC Trials

We extracted information on the baseline characteristics of PE patients including age, gender, creatinine clearance, causes of PE (unprovoked, cancer, or temporary risk factors), previous VTE, and anatomical extent of PE from the published DOAC trials. 6 7 8 14 In the AMPLIFY study, separate data for the PE cohort regarding age, gender, creatinine clearance, causes of PE, and previous VTE were not provided. 7 Information on the anatomical extent of PE was unavailable for the RE-COVER and RE-COVER II studies. 14 For the other three studies, limited extent was defined as involvement of 25% or less of the vasculature of a single lobe, whereas intermediate extent was defined as involvement of more than 25% of the vasculature of a single lobe or multiple lobes with involvement of 25% or less of the entire vasculature. The definition of extensive disease varied; it was defined as involvement of multiple lobes with compromise of 25% or more of the entire vasculature in EINSTEIN-PE and Hokusai-VTE, 6 8 whereas in AMPLIFY, extensive disease was defined as involvement of two or more lobes compromising 50% or more of the vasculature of each lobe. 7 The latter definition may classify more patients as having extensive disease than the former, because it focuses only on the vasculature of the involved lobes rather than the entire vasculature of the lungs.

### Objective 3: Anatomical Extent, Right Ventricular Dysfunction, Causes of PE, and Recurrent VTE in the Hokusai-VTE Study

The Hokusai-VTE study was a randomized, double-blind trial that compared heparin/edoxaban with heparin/warfarin in 8,292 acute, symptomatic VTE patients (ClinicalTrials.gov identifier: NCT00986154). 8 12 This analysis focuses on the 3,319 patients with PE. Initial treatment consisted of open-label enoxaparin or unfractionated heparin for at least 5 days followed by either edoxaban or warfarin for a minimum of 3 months and a maximum of 12 months. The dose of edoxaban was 60 mg once daily; this was reduced to 30 mg in patients with a creatinine clearance of 30 to 50 mL/minute, a body weight 60 kg or less, or in those receiving potent concomitant P-glycoprotein inhibitors such as verapamil or quinidine. Warfarin was started concomitantly with heparin and the heparin was stopped when the INR was 2.0 or higher. The primary efficacy outcome was the incidence of symptomatic recurrent VTE defined as a composite of deep vein thrombosis (DVT) or nonfatal or fatal PE over the 12 months of the study. This outcome was assessed by a central adjudication committee whose members were unaware of the treatment allocation. 8

In patients with PE, RVD was defined as a NT-proBNP level of 500 ng/mL or higher. Serum NT-proBNP levels were measured in samples collected at enrolment. Assays were performed centrally in Quintiles Laboratories (Marietta, Georgia, United States) using the Elecsys NT-proBNP electrochemiluminescence kit on the Roche Cobas e411 platform. Data on PE extent were not available for patients with NT-proBNP levels of less than 500 ng/mL or patients who did not have NT-proBNP levels measured. Causes of PE were classified as either unprovoked or associated with cancer or temporary risk factors (i.e., recent surgery, trauma, immobilization, or use of estrogen). 8 The definitions of cancer used in the studies were accepted.

In the current analysis, we correlated the anatomical extent of PE at baseline with NT-proBNP levels, causes of PE, and recurrent VTE at 12 months of follow-up. The results are presented as number of events and percentages.

## Results

### Rates of Recurrent VTE with or without Heparin Lead-in in PE Patients

**Fig. 1:**
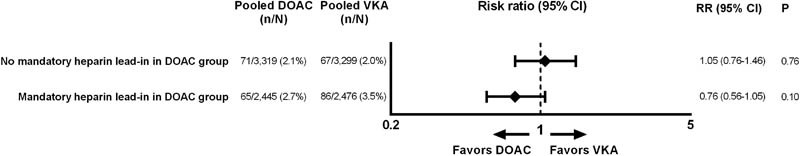
Relative risk of recurrent venous thromboembolism with or without a heparin lead-in. DOAC, direct oral anticoagulant; VKA, vitamin K antagonist.

Fig. 1 presents the pooled analyses of results that included 11,539 PE patients. Rates of recurrent VTE in patients treated with DOACs were compared with those in patients given standard-of-care treatment. The RR of recurrent VTE with DOAC treatment versus heparin/VKA was 0.76 (95% CI: 0.56–1.05; heterogeneity *I* ^2^ = 0%; recurrence rates 2.7 vs. 3.5%) with heparin lead-in and 1.05 (95% CI: 0.76–1.46; heterogeneity *I* ^2^ = 0%; recurrence rates 2.1 vs. 2.0%) without heparin lead-in.

### Patient Characteristics across the DOAC Trials

Table 1 details the similarities and differences in patient characteristics across the studies based on the published data. Three studies (RE-COVER, RE-COVER II, and Hokusai-VTE) had a mandatory heparin lead-in in the DOAC arm. Age and gender were comparable across the studies. The anatomical extent of PE was not determined in the RE-COVER studies. Although the Hokusai-VTE and EINSTEIN-PE studies used an identical definition of anatomical extent, almost 50% of the patients had extensive PE in the Hokusai-VTE study, whereas a quarter of the patients had extensive PE in the EINSTEIN-PE study. In the AMPLIFY study, approximately 35% had extensive PE using a different definition. Another difference was the proportion of patients with temporary risk factors, ranging from approximately 16% in RE-COVER to around 40% in EINSTEIN-PE.

**Table 1:** Characteristics of pulmonary embolism patients in the phase 3 trials evaluating direct oral anticoagulants

Characteristics	RE-COVER study 14		EINSTEIN-PE study 6		AMPLIFY study 7		Hokusai-VTE study 8	
	Dabigatran *N* = 795	Warfarin *N* = 807	Rivaroxaban *N* = 2,419	Warfarin *N* = 2,413	Apixaban *N* = 930	Warfarin *N* = 906	Edoxaban *N* = 1,650	Warfarin *N* = 1,669
Study design	Randomized, double-blind		Randomized, open label		Randomized, double-blind		Randomized, double-blind	
Heparin lead-in	LMWH or UFH ≥5 d	LMWH or UFH ≥5 d	No a	Enoxaparin ≥5 d	No a	Enoxaparin ≥5 d	LMWH or UFH ≥5 d	LMWH or UFH ≥5 d
PE population vs. total study population, *n* / *N* (%)	795/2,553 (31)	807/2,554 (32)	100%	100%	930/2,609 (36)	906/2,635 (34)	1,650/4,118 (40)	1,669/4,122 (40)
Age, mean ± SD	56 ± 16	56 ± 16	58 ± 7	58 ± 7	NA	NA	57 ± 17	57 ± 17
Male sex, *n* (%)	420 (53)	431 (53)	1,309 (54)	1,247 (52)	NA	NA	863 (52)	875 (52)
PE extent, *n* (%)								
Limited	NA	NA	309 (13)	299 (12)	79 (9)	89 (10)	128 (8)	123 (7)
Intermediate			1,392 (57)	1,424 (59)	392 (42)	395 (44)	679 (41)	682 (41)
Extensive			597 (25)	576 (24)	357 (38)	326 (36)	743 (45)	778 (47)
Not assessable			121 (5)	114 (5)	102 (11)	96 (10)	100 (6)	86 (5)
Creatinine clearance ≥30 to ≤50 mL/min, *n* (%)	NA	NA	207 (9)	191 (8)	NA	NA	116 (7)	120 (7)
Causes of PE, *n* (%) b								
Unprovoked	608 (77)	637 (78)	1,566 (65)	1,551 (64)	NA	NA	1,047 (64)	1,042 (61)
Cancer	52 (6)	43 (6)	114 (5)	109 (5)			169 (10)	188 (11)
Temporary risk factor	135 (17)	127 (16)	1,006 (41)	1,001 (41)			477 (29)	485 (29)
Previous VTE, *n* (%)	183 (23)	173 (21)	455 (19)	489 (20)	NA	NA	368 (17)	322 (19)
Treatment duration	6 mo		3, 6, or 12 mo		6 mo		3, 6, or 12 m	
Follow-up duration	6 mo		3, 6, or 12 mo c		6 mo		12 mo d	

Abbreviations: IQR, interquartile range; LMWH, low-molecular-weight heparin; NA, not available; PE, pulmonary embolism; SD, standard deviation; UFH, unfractionated heparin; VTE, venous thromboembolism.

a Median length of heparin treatment in the DOAC trials without mandatory heparin lead-in; EINSTEIN-PE study: all patients received ≥24 hours of LWMH treatment, and 35% ≥48 hours; AMPLIFY study: no LWMH treatment in 14% of patients and >70% received >12–24 hours of treatment.

b Patients could have multiple causes for pulmonary embolism.

c Dependent on the treatment duration.

d All patients followed up for 12 months regardless of treatment duration.

### Associations of Anatomical Extent with Right Ventricular Dysfunction, Causes of PE, and Recurrent VTE in the Hokusai-VTE Study

Of the 3,319 PE patients in the Hokusai-VTE study, 90% had NT-proBNP levels measured as baseline (1,484/1,650 in the heparin/edoxaban and 1,505/1,669 in the heparin/warfarin group). In patients receiving heparin/edoxaban, 465 of 1,650 (28%) had NT-proBNP levels of 500 ng/mL or higher compared with 507 of 1,669 (30%) in the heparin/warfarin arm; 1,019 of 1,650 (62%) of heparin/edoxaban and 998 of 1669 (60%) of heparin/warfarin recipients had NT-proBNP levels of less than 500 ng/mL. There was an association between anatomical extent of PE and elevated levels of NT-proBNP in both treatment arms; the proportion of patients with NT-proBNP levels of greater than 500 ng/mL was higher in patients with an intermediate and highest in patients with extensive PE compared with those with limited disease ( Table 2 ).

**Table 2:** Correlation between anatomical extent of PE and elevated NT-proBNP levels

Characteristics	Hokusai-VTE study	
	Heparin/Edoxaban *N* = 465	Heparin/Warfarin *N* = 507
PE extent for patients with NT-proBNP ≥500 ng/mL, *n* (%)		
Limited	18 (4)	22 (4)
Intermediate	133 (29)	136 (27)
Extensive	293 (63)	327 (65)
Not assessable	21 (4)	22 (4)

Abbreviations: NT-pro BNP, N-terminal pro-brain natriuretic peptide; PE, pulmonary embolism; VTE, venous thromboembolism.

The proportion of patients with unprovoked PE was comparable at approximately 60% in all three categories of anatomical extent, with similar proportions in the two treatment arms ( Table 3 ).

**Table 3:** Anatomical extent of PE and the proportion of patients with unprovoked PE

PE extent and unprovoked PE	Hokusai-VTE study	
	Heparin/Edoxaban *N* = 1,650	Heparin/Warfarin *N* = 1,669
Unprovoked PE per category of PE extent, *n* / *N* (%)		
Limited	75/128 (59)	72/123 (59)
Intermediate	415/679 (61)	422/682 (62)
Extensive	489/743 (66)	494/778 (64)
Not assessable	68/100 (68)	54/86 (63)

Abbreviation: PE, pulmonary embolism; VTE, venous thromboembolism.

In the edoxaban group, there was an association between anatomical extent of PE and recurrence rate, increasing from 1.6% with limited extent to 3.2% with extensive disease. This correlation was less clear in the heparin/warfarin group, because the recurrence rates in patients with intermediate and extensive PE were similar ( Table 4 ). In fact, the risk reduction with edoxaban appears consistent across the categories of anatomical extent.

**Table 4:** Correlation between anatomical extent of PE and recurrence

Recurrence rates	Hokusai-VTE study	
	Heparin/Edoxaban *N* = 1,650	Heparin/Warfarin *N* = 1,669
VTE recurrence rate per PE extent, *n* / *N* (%)		
Limited	2/128 (1.6)	3/123 (2.4)
Intermediate	17/679 (2.5)	27/682 (4.0)
Extensive	24/743 (3.2)	30/778 (3.9)
Not assessed	4/100 (4.0)	5/86 (5.8)

Abbreviations: PE, pulmonary embolism; VTE, venous thromboembolism.

## Discussion

Management of PE patients remains challenging, but this study provides new insights that could aid in guiding treatment decisions. There are three main findings. First, the indirect meta-analysis comparing DOAC and heparin/VKA therapy suggests a possible benefit for a heparin lead-in before starting the DOAC, because even though the CIs overlap, the point estimate with a heparin lead-in is lower. Another observation is that the incidence of VTE recurrence was 3.5% in the control arm of the studies where a heparin lead-in was used with the DOACs, whereas it was 2.0% in the control arm in the other comparison, suggesting that PE patients at higher risk of recurrence were enrolled in the heparin lead-in studies. The dilemma of whether or not to use heparin before starting a DOAC can only be resolved with a head-to-head comparison.

Second, the cross-study comparison of the PE patients enrolled in the DOAC studies reveals similarities among many of the baseline characteristics such as age, gender, and the proportion of patients with previous VTE. The most notable difference is the proportion of patients with extensive PE at the time of diagnosis, with 45% of patients in Hokusai-VTE having extensive PE compared with 25% in EINSTEIN-PE. A potential explanation for this difference might be the heparin lead-in, because physicians may have been more willing to enroll patients with extensive PE in a study that used a heparin lead-in in all patients.

Third, the in-depth analysis of the Hokusai-VTE study reveals an association between the anatomical extent of PE and elevated NT-proBNP levels, a known marker of RVD. 15 16 Only 4% of patients with limited disease had an elevated NT-proBNP, whereas the proportion with an elevated NT-proBNP was 29% with intermediate and 65% with extensive disease, findings indicative of a biological gradient. The risk of recurrence was almost twofold higher in patients with extensive disease than in those with limited disease in both treatment arms. Hence, the more patients with extensive disease, the greater the likelihood that the population studied has RVD, a known indicator of an increased risk of recurrent VTE and mortality. 17 18 19 20 Previous studies correlated PE extent to right ventricular enlargement, 21 and to thrombus load on CT with degree of right ventricular enlargement. 22 However, to our knowledge, our study is the first to investigate a correlation between anatomical extent of PE and NT-proBNP levels, and recurrent VTE events. Another novel finding is that the proportion of patients with unprovoked PE was comparable in all groups of anatomical extent, suggesting that the extent of the PE is independent of whether the PE is provoked or unprovoked.

A few aspects of the present analyses require comment. The results come from three different approaches: an indirect meta-analysis, a cross-study comparison, and a further in-depth analysis of data from the Hokusai-VTE study. However, all of the data come from well-executed, randomized, controlled trials. The results of the meta-analysis need to be interpreted with caution, because they are based on an indirect comparison. In addition, the observed difference in the point estimate in recurrent VTE between the two approaches (with or without heparin) may also be due to unknown differences between the study populations. Furthermore, an informal analysis of heparin lead-in among patients with DVT only appeared not to support a potential benefit of initial heparin (data not shown). Nonetheless, the findings are intriguing and require further investigation. Another remark is that since the Hokusai-VTE study was the last of the phase 3 DOAC trials, it had the advantage of taking some methodological issues from the previous studies a priori into account. The Hokusai-VTE study was the first to prospectively collect patient data on right ventricular dysfunction, as assessed by NT-proBNP levels and right ventricular/left ventricular (RV/LV) diameter ratio on CT, whereas the other studies did not. NT-proBNP levels were measured at the time of enrolment in the study, whereas anatomical extent of PE was measured at the time of diagnosis, and although the time between the diagnosis and inclusion in the study was only a couple of hours in the majority of patients, this may have influenced the findings.

In conclusion, we show an association between anatomical extent of PE, RVD, and the risk of recurrence. Because the indirect meta-analysis suggests a possible benefit of a heparin lead-in, future DOAC studies evaluating the role of heparin might focus on PE patients with more extensive disease.
